# Nuciferine Inhibits Proinflammatory Cytokines via the PPARs in LPS-Induced RAW264.7 Cells

**DOI:** 10.3390/molecules23102723

**Published:** 2018-10-22

**Authors:** Chao Zhang, Jianjun Deng, Dan Liu, Xingxia Tuo, Yan Yu, Haixia Yang, Nanping Wang

**Affiliations:** 1 Department of Nutrition and Food Safety, College of Public Health, Xi’an Jiaotong University, Xi’an 710061, China; zhangchao9277@163.com (C.Z.); liudan940305@163.com (D.L.); yyyyyy_214@163.com (X.T.); yuyan@mail.xjtu.edu.cn (Y.Y.); 2 Cardiovascular Research Center, Xi’an Jiaotong University, Xi’an 710061, China; 3 Shaanxi Key Laboratory of Degradable Biomedical Materials, School of Chemical Engineering, Northwest University, Xi’an 710069, China; dengjianjun@nwu.edu.cn; 4 The Advanced Institute for Medical Sciences, Dalian Medical University, Dalian 116044, China

**Keywords:** nuciferine, inflammation, PPARs, IL-6, TNF-α

## Abstract

Inflammation is important and has been found to be an underlying cause in many acute and chronic human diseases. Nuciferine, a natural alkaloid containing an aromatic ring, is found in the *nelumbo nucifera* leaves. It has been shown to have potential anti-inflammatory activities, but the molecular mechanism has remained unclear. In this study, we found that nuciferine (10 μM) significantly inhibited the lipopolysaccharide (LPS)-induced inflammatory cytokine IL-6 and TNF-α production in RAW 264.7 cells. In addition, the luciferase reporter assay results of different subtypes of the peroxisome proliferator-activated receptor (PPAR) showed that nuciferine dose-dependently activated all the PPAR activities. Specific inhibitors of PPARα and PPARγ significantly abolished the production of inflammatory cytokines as well as IκBα degradation. However, PPARδ inhibitor did not show this effect. Our results suggested a potential molecular mechanism of the anti-inflammatory effects of nuciferine in LPS-induced inflammation, at least in part, by activating PPARα and PPARγ in RAW 264.7 cells.

## 1. Introduction

Inflammatory responses are widely implicated in vast kinds of acute and chronic human diseases, including cancer, atherosclerosis, and diabetes [1]. Macrophages play a critical role and are involved in the self-regulating cycle of inflammation, as macrophages produce multiple pro-inflammatory cytokines and mediators that are involved in inflammation, such as the TNFα and the IL-6 [2]. Interference therapy that target macrophages and related cytokines may be some new approaches for controlling inflammatory diseases.

Regulation of the inflammatory response depends on a variety of potential mechanisms, including peroxisome proliferator-activated receptors (PPARs) actions [3]. PPARs are activated by their synthetic or natural ligands/modulators, which lead to the PPARs to bind to their specific DNA response elements, as heterodimers, with the retinoid X receptor (RXR) [4]. PPARs have been found to have three subtypes, which are named PPARα, PPARβ/δ, and PPAR. They play crucial roles in the regulation of lipid and glucose metabolism. In addition, accumulating evidence reveals that activation of the PPARs are involved in the various types of inflammatory processes, due to the inhibition of pro-inflammatory genes expression and negative regulation of pro-inflammatory transcription factor signaling pathways, in inflammatory cells [5]. Furthermore, activation of PPARs shows the anti-inflammatory effect by inhibiting the activation of nuclear factor-κB (NF-κB), leading to a decrease of pro-inflammatory cytokines and mediators [6]. Therefore, PPARs have been shown to be the drug targets to treat various related inflammatory diseases, such as vascular diseases, cancer, and neurodegenerative diseases [7]. Searching for the effective ligands or modulators of PPARs, for the prevention and clinical therapeutic options, is of great interest.

The natural product nuciferine ((*R*)-1,2-dimethoxyaporphine; Nuci) is an alkaloid found within the leaves of *Nymphaea caerulea* and *Nelumbo nucifera*, which is widely planted in Asia, the Middle East, and some countries in Africa [8]. Especially in China, lotus leaves are usually commercially available for tea due to its pharmacologic effects, such as losing weight, heat-clearing, and detoxifying, according to the traditional theory of Chinese medicine [9]. Recent studies showed that nuciferine, an important component of lotus leave extracts, can improve hepatic lipid metabolism [10], increase the glucose consumption, and stimulate insulin secretion [11]. Anti-inflammation activity of nuciferine was also reported in potassium oxonate-induced kidney inflammation [12], as well as Fructose-induced inflammatory responses [13], in vivo. However, the underlying molecular mechanisms of its anti-inflammatory effects are not fully understood. Based on the inflammatory-related functions of PPARs and the differences of the distinct tissue-specific expression, physiology, and ligand specificity of the PPARα, PPARβ/δ, and the PPARγ, the aim of this study was to investigate the effect of nuciferine on inflammation in lipopolysaccharide (LPS)-induced RAW264.7 cells and to observe if this effect is mediated by the three PPAR subtypes.

## 2. Results

### 2.1. Cytotoxicity of Nuciferine on RAW264.7 Cells

**Figure 1:**
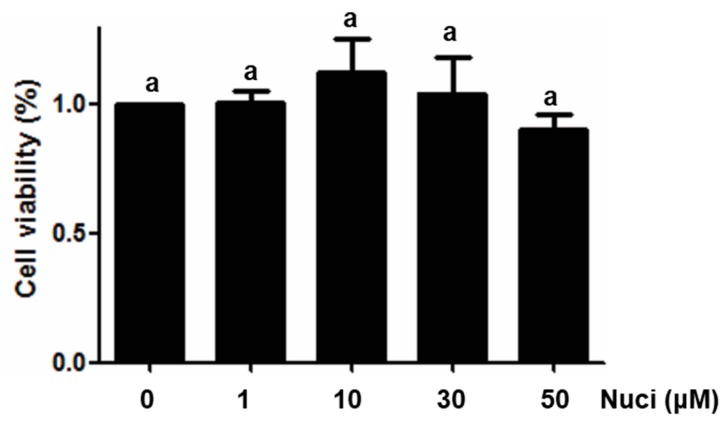
Cytotoxicity of Nuciferine on macrophage RAW264.7 cells. Cell viabilities of RAW264.7 cells treated with Nuciferine (0, 1, 3, 10 or 50 μM), for 24 h, were measured by MTT assay.

To test the effect of nuciferine on the cell viabilities of RAW264.7 cells, 3-(4,5-dimethyl-2-thiazolyl)-2,5-diphenyltetrazolium bromide (MTT) assay was performed in RAW264.7 cells, using different concentrations of nuciferine, ranging from 1 to 50 μM. After treatment for 24 h, cell viabilities were measured, and the results are shown in Figure 1. Compared with the control group (without nuciferine), no significant difference (*p* > 0.05) were found between control and all the treatment groups, indicating that nuciferine had no direct cytotoxicity, in this cell line. To avoid using a concentration of nuciferine higher than the normal physiological concentration, 10 μM was used in all of the following experiments.

### 2.2. Nuciferine Inhibited IL-6 and TNFα Production in LPS-Induced RAW264.7 Cells

**Figure 2:**
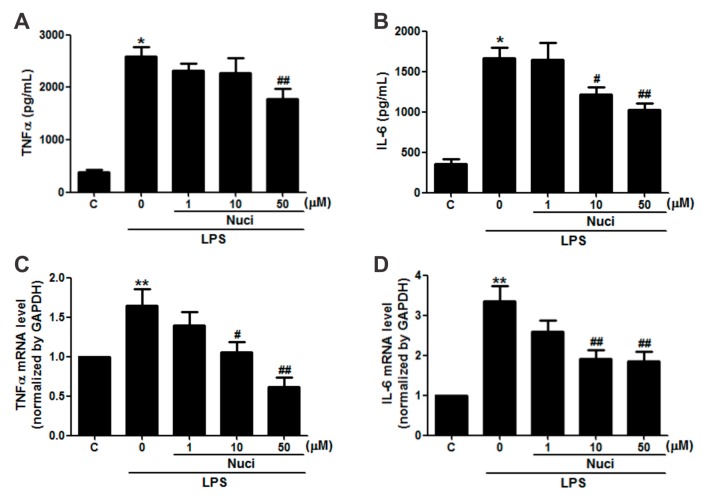
Nuciferine inhibits the LPS-induced TNFα and IL-6 production in RAW264.7 cells. RAW 264.7 cells were pretreated with nuciferine (0, 1, 10 or 50 μM for 24 h) and then stimulated with the LPS (500 ng/mL for 12 h), with a nuciferine withdrawal. (**A**,**B**) TNFα and IL-6 releases and (**C**,**D**) mRNA level of TNFα and IL-6, respectively. * *p* < 0.05 ** *p* < 0.01 vs. control, ^#^ *p* < 0. 05 ^##^ *p* < 0.01 vs. LPS treatment.

In order to evaluate whether the nuciferine has potential anti-inflammatory activity in RAW264.7 cells, the cells were pretreated with nuciferine (1~50 µM), for 24 h, before exposure to LPS, and the pro-inflammatory cytokines IL-6 and TNFα were examined (Figure 2). Without the LPS stimulation, the concentrations of TNFα and IL-6, in the cell medium, by ELISA were 380.5 ± 51.3 pg/mL and 352.1 ± 60.1 pg/mL, respectively (Figure 2A,B). The LPS treatment significantly increased (*p* < 0.05) both TNFα and IL-6 levels by 679.2% and 472.6%, respectively. Importantly, the nuciferine decreased both these cytokine levels induced by the LPS, in a dose-dependent manner. Meanwhile, gene expression of these cytokines, by RT-qPCR, showed the same trends as the ELISA results (Figure 2C,D). Pearson correlation analysis of both the protein and the mRNA levels of TNFα and IL-6, with the nuciferine concentrations, are shown in Table 1. With the LPS treatment, it showed significant negative correlation of the ratio of nuciferine concentration versus IL-6 protein level (Pearson *r* = −0.62, *p* = 0.004, *n* = 20) or mRNA level (Pearson *r* = −0.50, *p* = 0.02, *n* = 20). Similarly, significant negative correlations were found between nuciferine concentration and TNFα protein level (Pearson *r* = −0.58, *p* = 0.02, *n* = 20) or mRNA level (Pearson *r* = −0.69, *p* = 0.01, *n* = 20). All these results indicated that nuciferine had a potential anti-inflammatory effect, by reducing inflammatory cytokines production.

### 2.3. Nuciferine Increased the PPARs Activity

**Figure 3:**
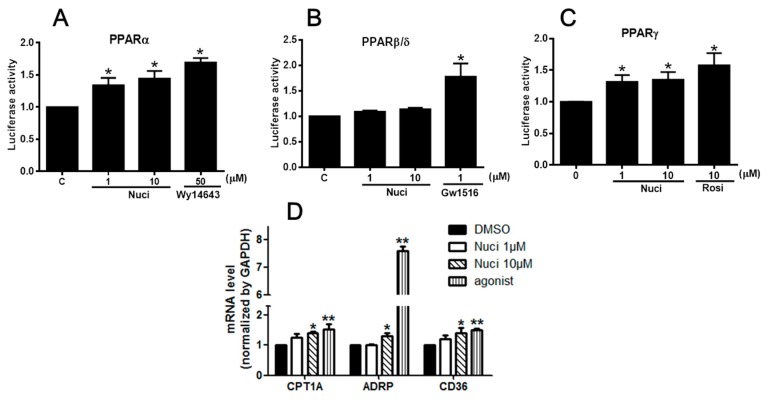
Effect of Nuciferine on PPARs transcription activities. (**A**–**C**) Luciferase reporter assay in RAW264.7 cells. (**D**) The relative mRNA expression of PPARs target genes. * *p* < 0.05 ** *p* < 0.01 vs. control.

PPARs are involved in the various types of inflammatory processes [5]. To study the potential molecular mechanism of nuciferine on the LPS-induced inflammation, we examined the effects of nuciferine on PPARs activity by a luciferase reporter assay. The cells were transfected with PPARs isoforms (PPARα/PPARδ/PPARγ) plasmid and reporter plasmid, followed by the treatment of nuciferine (1 or 10 µM) or PPARs agonists, for 24 h. All the selective agonists WY14643 for PPARα, GW501516 for PPARβ/δ, and rosiglitazone (Rosi) for PPARγ, significantly increased the fluorescence signal in the both the RAW264.7 cells and HEK 293 cells, confirming that the systems for detecting PPARs transactivation activity were correct. In the RAW 264.7 cells, nuciferine significantly increased the transcriptional activities of PPARα and PPARγ, in a dose-dependent manner, compared to control group. However, it did not affect PPARβ/δ activity (Figure 3A–C). Pearson correlation analysis (Table 2) shows a significant positive correlation of the ratio of nuciferine concentration, versus the PPARα activity (Pearson *r* = 0.70; *p* = 0.004, *n* = 15) and a significant positive correlation of the ratio of nuciferine concentration versus the PPARγ activity (Pearson *r* = 0.51; *p* = 0.05, *n* = 15). However, there was no significant correlation of the ratio of nuciferine concentration versus the PPARβ/δ activity (Pearson *r* = 0.29; *p* = 0.11, *n* = 15). Meanwhile, the luciferase reporter assay was carried out using the HEK 293 cells (Supplementary Figure S1) to verify the results. All the results showed that nuciferine increased the activities of the three PPARs but only significantly increased the PPARα and PPARγ activity. The mRNA levels of the targets genes of PPARs, such as carnitine palmitoyltransferase 1A (CPT-1A) for PPARα [14], adipose differentiation related protein (ADRP) for PPARβ/δ [15], CD36 for PPARγ [16], were further investigated (Figure 3D). All these target genes expressions were up-regulated by nuciferine, at 10 μM, in the RAW264.7 cells. Together, these results indicated that nuciferine could enhance PPARs transcriptional activity in mononuclear macrophages.

### 2.4. Antagonists of PPARα and PPARγ Abolished the Anti-Inflammatory Effects of Nuciferine

**Figure 4:**
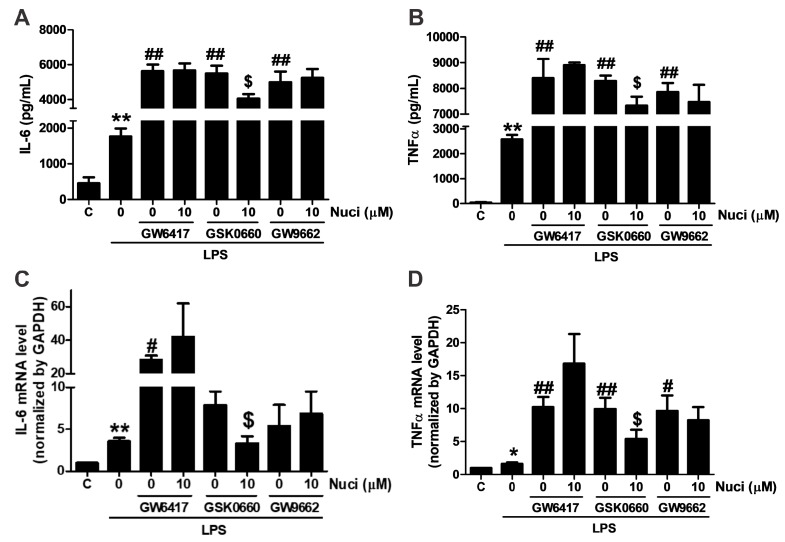
Antagonist of PPARα and PPARγ abolished the effects of nuciferine on the LPS-induced TNFα and IL-6 production. RAW264.7 cells were pretreated with GW6417/GSK0660/GW9662, for 12 h, followed by the nuciferine incubation, for 24 h, and then stimulated with LPS for 12 h. (**A**,**B**) production of the IL-6 and the TNFα in a cell medium supernatant. (**C**,**D**) mRNA expression of IL-6 and TNFα. * *p* < 0.05 ** *p* < 0.01 vs. control. ^#^ *p* < 0.05, ^##^ *p* < 0.01 vs. LPS treatment. ^$^ *p* < 0.05 vs. antagonist pretreatment followed by the LPS stimulation.

To further clarify whether the anti-inflammatory effect of nuciferine are mediated by PPARs, antagonists GW5417 for PPARα, GSK0660 for PPARβ/δ, GW9662 for PPARγ were co-administrated with nuciferine, for 24 h, in the LPS-induced RAW264.7 cells, respectively (Figure 4). As the results before, when stimulated with the LPS, the content of IL-6 and TNFα were increased. All these antagonists increased the pro-inflammatory cytokines, compared with the group treated with the LPS only. However, the effect of nuciferine was abolished in the presence of the PPARα and PPARγ antagonists, indicating that the anti-inflammatory effect of nuciferine, at least partially, went through the PPARs activation.

### 2.5. Nuciferine Decreased LPS Induced IκB-α Degradation through PPARs Activition

**Figure 5:**
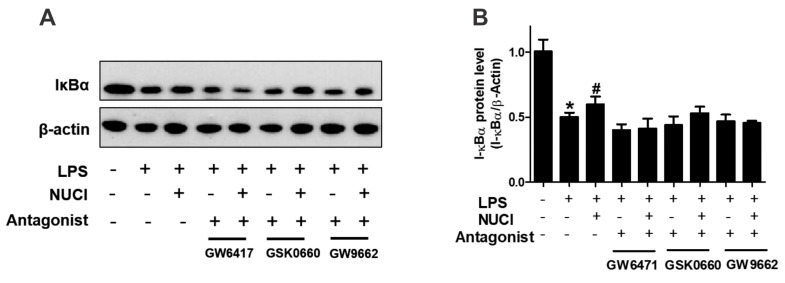
Anti-inflammatory effects of nuciferine on the LPS-induced inflammatory response, are PPARs dependent. RAW264.7 cells were pretreated with GW6417/GSK0660/GW9662, for 12 h, followed by the Nuciferine incubation and then stimulated by the LPS with a nuciferine withdrawal. Levels of the expression of IκBα were detected by Western blotting. * *p* < 0.05 vs. control group; ^#^ *p* < 0.05 vs. LPS group.

PPARs exert anti-inflammatory effects by regulating the NF-κB signal pathway. To further investigate the mechanisms of nuciferine on anti-inflammatory effect, the degradation of IκB-α protein levels were determined with exposure to the nuciferine in the LPS-treated RAW 264.7 cells (Figure 5, original results see Figure S2). When stimulated only with LPS, the cytosolic IκB-α protein was markedly degraded, consistent with the THP-1 treatment results [17]. However, nuciferine treatment attenuated the pro-inflammatory effect of the LPS. Furthermore, the effects of nuciferine were abolished by a co-incubation with PPARs antagonists. The results suggested that nuciferine dramatically inhibited the LPS-induced NF-κB activation and its effect was PPARs-dependent.

## 3. Discussion

Our present study has shown that treatment with nuciferine ameliorates the LPS-induced inflammation in RAW264.7 cells. Importantly, it was found that the protective effect of nuciferine is mediated by PPARs activation. These results highlight the potential use of nuciferine for preventing inflammation.

Overexpression of the inflammatory mediators is closely associated with systemic injury. There is evidence that anti-inflammatory treatment has become an important component of inflammatory diseases [18]. Inflammatory mediator inhibitors can be shown to have beneficial effects in improving the severity of inflammation-related diseases. A wide variety of phytochemicals derived from natural plant have anti-inflammatory effects, such as phenolics, terpenolids, and alkaloids [19]. Nuciferine, an alkaloid found in the lotus leaves, exerted a protective effect against inflammation, in vivo [20] and in vitro [21]. Our results showed that nuciferine decreased the expression of inflammatory cytokines IL-6 and TNFα, in both protein and gene levels, dose dependently, in the LPS-treated RAW264.7 cells, indicating that nuciferine had potential anti-inflammatory effects.

Nuciferine, a natural alkaloid from the lotus leaves, have been reported to exert multiple beneficial effects, in vivo and in vitro, such as anti-tumor [22] and insulin stimulatory effects [23]. Our recent results showed that nuciferine improved the hepatic steatosis in high-fat diet/streptozocin-induced diabetic mice [24]. Some studies reported that nuciferine suppressed the inflammation by regulating inflammatory signaling through different signal pathways. For example, in hyperuricemia mouse model, nuciferine inhibited renal inflammation through suppression of Toll-like receptor 4/myeloid differentiation factor 88/NF-κB signaling and a NOD-like receptor family, pyrin domain containing 3 (NLRP3) inflammasome [12]. Similarly, in vitro studies, nuciferine exerted the anti-inflammatory and antilipemic effects, as well as the siRNA Per-Arnt-Sim kinase treatment group in oleic acid-induced hepatic steatosis, in HepG_2_ cells, indicating a potential molecular pathway of the anti-inflammation effect of nuciferine [21]. As we know, the three subtypes of PPARs exert anti-inflammatory effects in vivo and in vitro by several different molecular mechanisms [25,26,27]. PPARα [28] and PPARγ [29] were shown to repress some other transcription factors, such as NF-κB signal pathway, to reduce the release of inflammatory cytokines including IL-6 and TNFα, when they were activated by their ligands. The anti-inflammatory effects of PPARβ are mediated by ligand-independent repression [30]. Owing to the anti-inflammatory effect of PPARs, we used the luciferase reporter assay and the target gene transcription of PPARα/PPARγ/PPARδ to test if the PPAR family is involved in the anti-inflammatory effect of nuciferine. The results showed that nuciferine activated the PPAR family, especially the PPARα and the PPARγ. Moreover, the antagonists of the PPAR family GW6417/GSK0660/GW9662 were treated in the cells to block the PPARs activities, before the nuciferine treatment. What’s interesting is that all the antagonist treatment increased the inflammation markers. The protective effect of nuciferine was remarkably diminished by the inhibition of PPARα and PPARγ, indicating that the anti-inflammatory effect of nuciferine, at least in part, went through the PPARs receptor activation. To confirm these results, the protein expression of the activated PPARs and the total PPARs should also be tested, using immunoblotting, in the further studies. Our recent in vivo results also clarified that nuciferine-activated PPARα in the liver tissues, in a diabetic mouse model [24]. Nuciferine is hydrophobic, consistent with the structures of most PPAR agonists. It could interact with the ligand-binding domain of PPARs, in theory, leading to the stabilization of the configuration of the hydrophobic core and subsequently the activation of PPARs to regulate the gene transcription [31]. However, more binding mechanisms between the nuciferine and the PPARs should be further studied.

It is well known that NF-κB is an important target for inflammatory therapeutic strategy [32]. PPARs have recently been shown to exert the anti-inflammatory activity by reducing the DNA-binding activity of NF-κB and suppressing its nucleus translocation, which attenuates the cytokine production and reduces tissue injury [32,33,34]. NF-κB is a crucial factor to activate the inflammatory genes transcription, including pro-inflammatory cytokines, such as TNFα and IL-6 [35]. In addition, IκBα expression was accompanied by a decrease in NF-κB DNA binding activity [36]. Our results showed that nuciferine treatment alters the IκBα cellular content in LPS stimulation. Moreover, the specific inhibitors for PPARs reversed the effect of nuciferine, partially or completely, indicating that nuciferine could prevent IκBα degradation via PPARs activation, under the LPS stimulated conditions.

Overall, our studies demonstrated that nuciferine, with a concentration of 10 μM, attenuated the LPS-induced inflammation through activation of PPARs, especially PPAR-α and-γ, in RAW264.7 cells. These findings suggest that nuciferine may be a potentially important candidate for inflammatory diseases.

## 4. Materials and Methods

### 4.1. Reagents

Nuciferine (purity by HPLC > 98.0%) was purchased from APP-CHEM (YHI-039, Xi’an, Shanxi, China). Dulbecco’s modified Eagle’s medium-high glucose (DMEM), fetal bovine serum (FBS), 3-(4,5-dimethylthiazol-2-yl)-2,5-diphenyltetrazolium bromide (MTT), and Lipofectamine 2000 reagent were purchased from Invitrogen (Carlsbad, CA, USA). Lipopolysaccharide (LPS), PPARs agonists WY14643, GW501516, rosiglitazone (Rosi) were purchased from Sigma (St. Louis, MO, USA). TRIzol reagent was purchased from Invitrogen (Carlsbad, CA, USA). IL-6 and TNF-α Mouse ELISA Kit was obtained from Elabscience Biotechnology Co. Ltd. (Wuhan, Hubei, China). Super Script II Rnase H Reverse Transcriptase kit was purchased from Invitrogen (Carlsbad, CA, USA).

### 4.2. Cell Culture

Murine macrophage RAW264.7 cells (ATCC, Rockville, MD, USA) and human embryonic kidney cells (HEK293 cells, ATCC, Rockville, MD, USA) were cultured with DMEM containing 10% FBS, 100 U/mL penicillin, and 100 U/mL streptomycin. Cells were maintained at 37 °C, in a humidified atmosphere of 5% CO_2_ and 95% air. RAW264.7 cells were seeded into plates and treated at approximately 80% confluence.

### 4.3. Cytotoxicity

RAW264.7 cells were seeded at a density of 1.5 × 10^3^ cells/well in 96-well plates. After 24 h, cells were treated with different concentrations of nuciferine (0–50 μM), for 24 h, followed by an addition 20 μL MTT solution (5 g/L), to each well, for 4 h. The insoluble formazan product was dissolved in 150 μL/well dimethyl sulfoxide (DMSO), after washing out the supernatant [37]. Then, the absorbance at 490 nm was measured using a microplate reader (Olympus America Inc., New York, NY, USA). The percentage of cytotoxicity was calculated by the equation: Cytotoxicity (%) = (1 − A_490_ of sample)/A_490_ of control well.

### 4.4. IL-6 and TNFα Levels Determination

RAW264.7 cells were grown into 12-well plates, treated with different concentrations of nuciferine (0, 1, 10 or 50 μM) and stimulated with LPS (500 ng/mL). Cell-free supernatants were collected and the levels of pro-inflammatory cytokines, TNFα and IL-6 were measured using ELISA kits, by a determination of the absorbance at 450 nm, according to the manufacturer’s instructions. Standard curves were used to calculate the concentration of TNFα and IL-6 in each sample.

### 4.5. PPARs Luciferase Reporter Assay

HEK293T cells and RAW264.7 cells were plated into 12-well plates at 4 × 10^5^ cells/well without antibiotics. After 24 h at 60% confluence, cells were transfected according to the manufacturer’s instructions. Briefly, PPARs isoforms (PPARα/PPARδ/PPARγ) plasmid (0.9 µg), reporter plasmid PPRE×3-TK-LUC (0.3 µg) and β-gal (0.1 µg) were transfected into the cells, using Lipofectamine 2000 reagents (1:1), for 4 h. Since transfection efficiency is typically low in RAW264.7 cells, more Lipofectamine 2000 was needed (1:2.5) and the transfection time was extended to 24 h. The medium was replaced with a complete media containing DMSO, nuciferine, or PPARs agonists, for 24 h. The cells were harvested and lysed to measure the luciferase activities using a luciferase assay kit, according to the manufacturer’s instructions. The β-gal was transfected to normalize the transfection efficiency [38].

### 4.6. RNA Isolation and Analysis

Cells were cultured into 12-well plate with a density of 4 × 10^5^ cells/well. Total RNA was isolated using TRIzol reagent and reverse transcribed into cDNA. Real-time quantitative polymerase chain reaction (RT-qPCR) was performed as described by Yang et al. [39]. Glyceraldehyde-3-phosphate dehydrogenase (*Gapdh*) was used as an internal control. Ct values of the sample were calculated, and the mRNA levels were analyzed by 2^−ΔΔCt^ method and normalized to *Gapdh* [40]. The primer sequences were listed in Supplemental Table S1.

### 4.7. Immunoblotting

RAW264.7 Cell lysates were prepared using a lysis buffer containing 0.1% Triton X-100 and proteinase inhibitors (Roche, Nutley, NJ, USA). Protein concentrations were determined using the BCA protein assay kit (Thermo Scientific, PA, USA). Western blot was performed as described by Yang et al. [39]. After blocking the membranes, primary rabbit antibody against IκBα (Santa Cruz Biotechnology, Dallas, TX, USA) was incubated with a ratio of 1:1000 overnight. β-actin (1:5000, Santa Cruz Biotechnology, Dallas, TX, USA) was used as a loading control. Membranes were then washed with TBST and incubated with the secondary antibodies conjugated to anti-rabbit or anti-mouse HRP-conjugated secondary antibodies (1:3000, Santa Cruz Biotechnology, Dallas, TX, USA) for 1 h. Bands were detected by enhanced chemiluminescence using ECL (Amersham Biosciences, Picataway, NJ, USA) and then visualized by X-ray films.

### 4.8. Data Statistics

Quantitative data are expressed as mean ± SEM using SPSS 18.0 (IBM Corporation, Chicago, IL, USA). Student *t* test and ANOVA followed by Tukey’s post hoc test were used to analyze the significant difference between two or more groups, respectively. The rank-based test methods were employed when data were not in a normal distribution or the variances were not homogeneous. All the results were representative of at least three independent experiments.

## Figures and Tables

**Table 1:** Nuciferine inhibits the LPS-induced TNFα and IL-6 production in RAW264.7 cells.

Nuciferine (μM)	LPS (ng/mL)	TNFα		IL-6	
		Protein (pg/mL)	mRNA	Protein (pg/mL)	mRNA
0	0	380.51 ± 51.27	0.99 ± 0.01	352.01 ± 60.02	1.01 ± 0.01
0	500	2584.46 ± 179.60 *	1.64 ± 0.21 **	1663.71 ± 137.20 *	3.34 ± 0.39 **
1	500	2315.98 ± 146.64	1.39 ± 0.17	1643.94 ± 209.69	2.59 ± 0.28
10	500	2139.87 ± 275.53	1.05 ± 0.13 ^#^	1216.91 ± 88.18 ^#^	1.92 ± 0.20 ^##^
50	500	1772.82 ± 203.58 ^##^	0.61 ± 0.11 ^##^	1028.78 ± 74.61 ^##^	1.85 ± 0.21 ^##^

Quantitative data are presented as mean ± SEM. * *p* < 0.05 ** *p* < 0.01 vs. control, ^#^ *p* < 0. 05 ^##^ *p* < 0.01 vs. LPS treatment.

**Table 2:** Effect of Nuciferine on PPARs transcription activities in RAW264.7 cells.

Nuci (μM)	PPARα	PPARβ/δ	PPARγ
0	1.03 ± 0.04	0.99 ± 0.01	1.02 ± 0.01
1	1.34 ± 0.11 *	1.03 ± 0.19	1.31 ± 0.09 *
10	1.44 ± 0.11 *	1.13 ± 0.27	1.35 ± 0.12 *
agonist	1.69 ± 0.06 *	1.17 ± 0.22 *	1.57 ± 0.13 *

Quantitative data are presented as mean ± SEM. * *p* < 0.05 vs. control.

## Supplementary Materials

The following are available online, Figure S1: Effect of Nuciferine on PPARs transcription activities in HEK 293 cells. Figure S2: Original western blot films of Figure 5A. Table S1: Sequences of primers for qRT-PCR.

## References

[1] Boteanu R.M., Suica V.I., Uyy E., Ivan L., Dima S.O., Popescu I., Simionescu M., Antohe F. (2017). Alarmins in chronic noncommunicable diseases: Atherosclerosis, diabetes and cancer. J. Proteomics.

[2] Laskin D.L., Pendino K.J. (1995). Macrophages and inflammatory mediators in tissue injury. Annu. Rev. Pharmacol. Toxicol..

[3] Moraes L.A., Piqueras L., Bishop-Bailey D. (2006). Peroxisome proliferator-activated receptors and inflammation. Pharmacol. Ther..

[4] Fan W., Evans R. (2015). PPARs and ERRs: Molecular mediators of mitochondrial metabolism. Curr. Opin. Cell Biol..

[5] Daynes R.A., Jones D.C. (2002). Emerging roles of PPARs in inflammation and immunity. Nat. Rev. Immunol..

[6] He X., Liu W., Shi M., Yang Z., Zhang X., Gong P. (2017). Docosahexaenoic acid attenuates LPS-stimulated inflammatory response by regulating the PPARgamma/NF-κB pathways in primary bovine mammary epithelial cells. Res. Vet. Sci..

[7] Flores-Bastías O., Karahanian E. (2018). Neuroinflammation produced by heavy alcohol intake is due to loops of interactions between Toll-like 4 and TNF receptors, peroxisome proliferator-activated receptors and the central melanocortin system: A novel hypothesis and new therapeutic avenues. Neuropharmacology.

[8] Sharma B.R., Gautam L.N., Adhikari D., Karki R. (2017). A Comprehensive Review on Chemical Profiling of Nelumbo Nucifera: Potential for Drug Development. Phytother. Res..

[9] Li Z., Liu J., Zhang D., Du X., Han L., Lv C., Li Y., Wang R., Wang B., Huang Y. (2018). Nuciferine and paeoniflorin can be quality markers of Tangzhiqing tablet, a Chinese traditional patent medicine, based on the qualitative, quantitative and dose-exposure-response analysis. Phytomedicine.

[10] Ma C., Li G., He Y., Xu B., Mi X., Wang H., Wang Z. (2015). Pronuciferine and nuciferine inhibit lipogenesis in 3T3-L1 adipocytes by activating the AMPK signaling pathway. Life Sci..

[11] Nguyen K.H., Ta T.N., Pham T.H., Nguyen Q.T., Pham H.D., Mishra S., Nyomba B.L. (2012). Nuciferine stimulates insulin secretion from beta cells-an in vitro comparison with glibenclamide. J. Ethnopharmacol..

[12] Wang M.X., Liu Y.L., Yang Y., Zhang D.M., Kong L.D. (2015). Nuciferine restores potassium oxonate-induced hyperuricemia and kidney inflammation in mice. Eur. J. Pharmacol..

[13] Wang M.X., Zhao X.J., Chen T.Y., Liu Y.L., Jiao R.Q., Zhang J.H., Ma C., Liu J.H., Pan Y., Kong L.D. (2016). Nuciferine Alleviates Renal Injury by Inhibiting Inflammatory Responses in Fructose-Fed Rats. J. Agric. Food Chem..

[14] Ohashi K., Munetsuna E., Yamada H., Ando Y., Yamazaki M., Taromaru N., Nagura A., Ishikawa H., Suzuki K., Teradaira R. (2015). High fructose consumption induces DNA methylation at PPARalpha and CPT1A promoter regions in the rat liver. Biochem. Biophys. Res. Commun..

[15] Zhao S., Kanno Y., Li W., Wakatabi H., Sasaki T., Koike K., Nemoto K., Li H. (2016). Picrasidine N Is a Subtype-Selective PPARβ/δ Agonist. J. Nat. Prod..

[16] Zhong Q., Zhao S., Yu B., Wang X., Matyal R., Li Y., Jiang Z. (2015). High-density lipoprotein increases the uptake of oxidized low density lipoprotein via PPARgamma/CD36 pathway in inflammatory adipocytes. Int. J. Biol. Sci..

[17] Bao W., Luo Y., Wang D., Li J., Wu X., Mei W. (2018). Sodium salicylate modulates inflammatory responses through AMP-activated protein kinase activation in LPS-stimulated THP-1 cells. J. Cell. Biochem..

[18] Wu Y., Xie G., Xu Y., Ma L., Tong C., Fan D., Du F., Yu H. (2015). PEP-1-MsrA ameliorates inflammation and reduces atherosclerosis in apolipoprotein E deficient mice. J. Transl. Med..

[19] Zhu F., Du B., Xu B. (2018). Anti-inflammatory effects of phytochemicals from fruits, vegetables, and food legumes: A review. Crit. Rev. Food Sci. Nutr..

[20] Wu H., Yang Y., Guo S., Yang J., Jiang K., Zhao G., Qiu C., Deng G. (2017). Nuciferine Ameliorates Inflammatory Responses by Inhibiting the TLR4-Mediated Pathway in Lipopolysaccharide-Induced Acute Lung Injury. Front. Pharmacol..

[21] Zhang D.D., Zhang J.G., Wu X., Liu Y., Gu S.Y., Zhu G.H., Wang Y.Z., Liu G.L., Li X.Y. (2015). Nuciferine downregulates Per-Arnt-Sim kinase expression during its alleviation of lipogenesis and inflammation on oleic acid-induced hepatic steatosis in HepG2 cells. Front. Pharmacol..

[22] Liu W., Yi D.D., Guo J.L., Xiang Z.X., Deng L.F., He L. (2015). Nuciferine, extracted from Nelumbo nucifera Gaertn, inhibits tumor-promoting effect of nicotine involving Wnt/β-catenin signaling in non-small cell lung cancer. J. Ethnopharmacol..

[23] Ma C., Wang J., Chu H., Zhang X., Wang Z., Wang H. (2014). Purification and characterization of aporphine alkaloids from leaves of Nelumbo nucifera Gaertn and their effects on glucose consumption in 3T3-L1 adipocytes. Int. J. Mol. Sci..

[24] Zhang C., Deng J., Liu D., Tuo X., Xiao L., Lai B., Yao Q., Liu J., Yang H., Wang N. (2018). Nuciferine ameliorates hepatic steatosis in high-fat diet/streptozocin-induced diabetic mice through a PPARalpha/PPARgamma coactivator-1alpha pathway. Br. J. Pharmacol..

[25] Adhikary T., Wortmann A., Schumann T., Finkernagel F., Lieber S., Roth K., Toth P.M., Diederich W.E., Nist A., Stiewe T. (2015). The transcriptional PPARβ/δ network in human macrophages defines a unique agonist-induced activation state. Nucleic Acids Res..

[26] Yang W., Rachez C., Freedman L.P. (2000). Discrete roles for peroxisome proliferator-activated receptor gamma and retinoid X receptor in recruiting nuclear receptor coactivators. Mol. Cell. Biol..

[27] Bougarne N., Paumelle R., Caron S., Hennuyer N., Mansouri R., Gervois P., Staels B., Haegeman G., De Bosscher K. (2009). PPARα blocks glucocorticoid receptor α-mediated transactivation but cooperates with the activated glucocorticoid receptor α for transrepression on NF-κB. Proc. Natl. Acad. Sci. USA.

[28] Azuma Y.T., Nishiyama K., Matsuo Y., Kuwamura M., Morioka A., Nakajima H., Takeuchi T. (2010). PPARalpha contributes to colonic protection in mice with DSS-induced colitis. Int. Immunopharmacol..

[29] Barish G.D., Atkins A.R., Downes M., Olson P., Chong L.W., Nelson M., Zou Y., Hwang H., Kang H., Curtiss L. (2008). PPARδ regulates multiple proinflammatory pathways to suppress atherosclerosis. Proc. Natl. Acad. Sci. USA.

[30] Yang H., Xiao L., Wang N. (2017). Peroxisome proliferator-activated receptor α ligands and modulators from dietary compounds: Types, screening methods and functions. J. Diabetes.

[31] Tak P.P., Firestein G.S. (2001). NF-κB: A key role in inflammatory diseases. J. Clin. Investig..

[32] Hernandez-Rodas M.C., Valenzuela R., Echeverria F., Rincon-Cervera M.A., Espinosa A., Illesca P., Munoz P., Corbari A., Romero N., Gonzalez-Manan D. (2017). Supplementation with Docosahexaenoic Acid and Extra Virgin Olive Oil Prevents Liver Steatosis Induced by a High-Fat Diet in Mice through PPAR-alpha and Nrf2 Upregulation with Concomitant SREBP-1c and NF-kB Downregulation. Mol. Nutr. Food Res..

[33] Silva-Veiga F.M., Rachid T.L., de Oliveira L., Graus-Nunes F., Mandarim-de-Lacerda C.A., Souza-Mello V. (2018). GW0742 (PPAR-β agonist) attenuates hepatic endoplasmic reticulum stress by improving hepatic energy metabolism in high-fat diet fed mice. Mol. Cell. Endocrinol..

[34] Sharma S., Sharma P., Kulurkar P., Singh D., Kumar D., Patial V. (2017). Iridoid glycosides fraction from Picrorhiza kurroa attenuates cyclophosphamide-induced renal toxicity and peripheral neuropathy via PPAR-gamma mediated inhibition of inflammation and apoptosis. Phytomedicine.

[35] Imanifooladi A.A., Yazdani S., Nourani M.R. (2010). The role of nuclear factor-κB in inflammatory lung disease. Inflamm. Allergy-Drug Targets.

[36] Delerive P., Gervois P., Fruchart J.C., Staels B. (2000). Induction of IkappaBalpha expression as a mechanism contributing to the anti-inflammatory activities of peroxisome proliferator-activated receptor-alpha activators. J. Biol. Chem..

[37] Tolosa L., Donato M.T., Gomez-Lechon M.J. (2015). General Cytotoxicity Assessment by Means of the MTT Assay. Methods Mol. Biol..

[38] Cheung S.T., Shakibakho S., So E.Y., Mui A.L.F. (2015). Transfecting RAW264.7 Cells with a Luciferase Reporter Gene. J. Visualized Exp..

[39] Yang H., Xiao L., Yuan Y., Luo X., Jiang M., Ni J., Wang N. (2014). Procyanidin B2 inhibits NLRP3 inflammasome activation in human vascular endothelial cells. Biochem. Pharmacol..

[40] Livak K.J., Schmittgen T.D. (2001). Analysis of relative gene expression data using real-time quantitative PCR and the 2^−ΔΔCT^ Method. Methods.

