# Supplementary material for: Nuciferine Inhibits Proinflammatory Cytokines via the PPARs in LPS-Induced RAW264.7 Cells
Source: Molecules. 2018 Oct 22;23(10):2723. doi: 10.3390/molecules23102723 (PMC6222486; doi:10.3390/molecules23102723)
Supplement: Supplementary file 1 [file molecules-23-02723-s001.pdf]

Supporting Information

Nuciferine Inhibits Proinflammatory Cytokines via the PPARs in LPS-Induced RAW264.7 Cells

Chao Zhang [1,2,†], Jianjun Deng [3,†], Dan Liu [1], Xingxia Tuo [1], Yan Yu [1], Haixia Yang [1,*], Nanping Wang [2,4,*]

[1] Department of Nutrition and Food Safety, College of Public Health, Xi'an Jiaotong University, Xi'an 710061, China, zhangchao9277@163.com (C.Z.); liudan940305@163.com (D.L.); yyyyyy_214@163.com (X.T.); yuyan@mail.xjtu.edu.cn (Y.Y.)

[2] Cardiovascular Research Center, Xi'an Jiaotong University, Xi'an 710061, Chinaf

[3] Shaanxi Key Laboratory of Degradable Biomedical Materials, School of Chemical Engineering, Northwest University, Xi'an 710069, China; dengjianjun@nwu.edu.cn (J.D.)

[4] The Advanced Institute for Medical Sciences, Dalian Medical University, Dalian 116044, China.

* Correspondence: yanghx@xjtu.edu.cn (H.Y.); nanpingwang2003@yahoo.com (N.P.); Tel.: +86-029-8265-5107 (H.Y.)

† These authors contributed equally to this work.

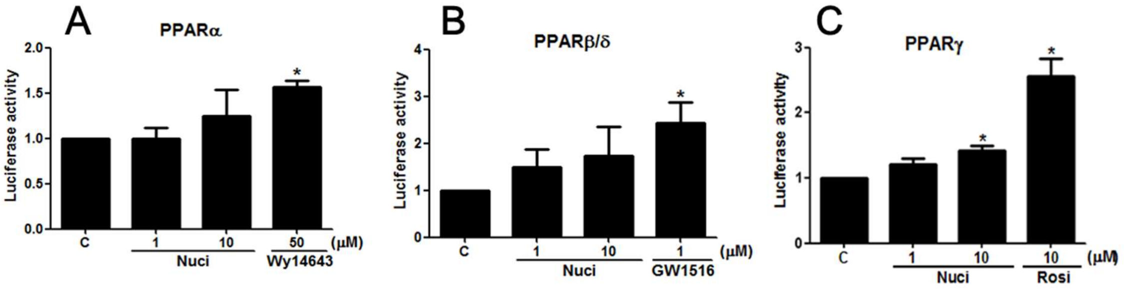

Figure S1. Effect of Nuciferine on PPARs transcription activities in HEK 293 cells.
* $p < 0.05$ ** $p < 0.01$ vs. control.

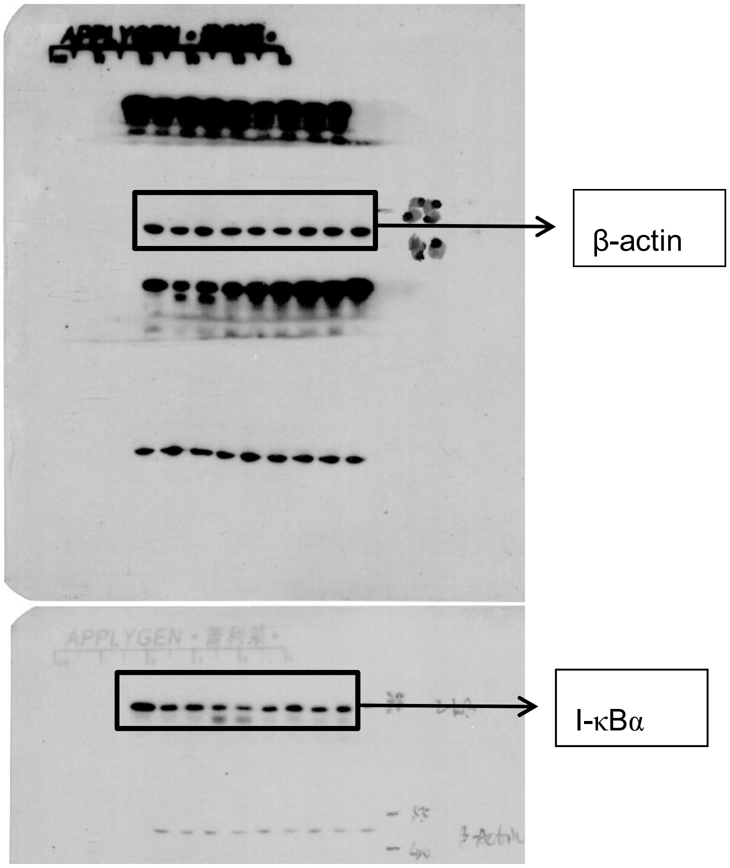

Figure S2. Original western blot films of Figure 5A.

Table S1. Sequences of primers for qRT-PCR

GENE	Forward Primer (5'-3')	Reverse Primer (5'-3')
GAPDH	ACCACAGTCCATGCCATCAC	TCCACCACCCTGTTGCTRTA
IL-6	TATGAACAGCGATGACCACTG	TTGCTCTGAATGACTCTGGCTT
TNFα	TCAGAGGGCCTGTACCTCAT	GGAAGACCCCTCCCAGATAG
CPT1-A	CTCAGTGGGAGCGACTCTTCA	GGCCTCTGTGGTACACGACAA
CD36	GCCTCCTTTCCACCTTTTGT	TCTGTACACGGGGATTCCTT
ADRP	CTGCTCACGAGCTGCATCATC	TGTGAGATGGCAGAGAACGGT
